# *TP53* Mutation Heterogeneity Refines Prognostic Stratification in *IDH*‐Wildtype Glioma

**DOI:** 10.1002/mco2.71041

**Published:** 2026-10-04

**Authors:** Kuilin Liao, Zhi Ye, Songtao Qi, Chang Xu, Yudi Huang, Zhiyong Li, Guozhong Yi, Yuou Qin, Qiuming Pan, Guiling Liu, Chao Chen, Zhuoyang Chen, Hongrui Li, Junxi Wang, Xi'an Zhang, Guanglong Huang, Shanqiang Qu

**Affiliations:** ^1^ Department of Neurosurgery Nanfang Hospital Southern Medical University Guangzhou Guangdong People's Republic of China; ^2^ Nanfang Glioma Center Nanfang Hospital Southern Medical University Guangzhou Guangdong People's Republic of China; ^3^ Institute of Brain disease Nanfang Hospital Southern Medical University Guangzhou Guangdong People's Republic of China; ^4^ Laboratory for Precision Neurosurgery Nanfang Hospital Southern Medical University Guangzhou Guangdong People's Republic of China; ^5^ The First Clinical School of Medicine Southern Medical University Guangzhou Guangdong People's Republic of China; ^6^ Department of Neurosurgery Ganzhou Hospital—Nanfang Hospital Southern Medical University Ganzhou Jiangxi People's Republic of China

**Keywords:** genomics, glioma, *IDH‐*wildtype, prognosis, *TP53* mutation

## Abstract

Clinically, *TP53* mutation (*TP53*‐mut) has long been simplistically dichotomized as mutant versus wildtype, overlooking their structural and functional complexity. The prognostic significance of *TP53*‑mut in *IDH*‑wildtype (*IDH*‑wt) gliomas remains controversial. In 237 *IDH*‑wt gliomas, we integrated clinical, genomic, and follow‑up data to test whether mutation subtypes refine risk stratification. In this cohort, 73 patients (73/237, 30.8%) harbored *TP53*‐mut, which was significantly associated with worse overall survival. Subgroup analysis further revealed that patients with mutations in the β‑strand region had a median outcome of only 12.0 months, markedly inferior to those with mutations in the α‑helix (22.5 months), loop (23.9 months), and turn/other regions (23.6 months). Multivariable Cox regression analysis showed that *TP53*‐mut was an independent prognostic factor associated with worse outcome (HR = 1.91; 95%CI: 1.29–2.84; *p = *0.001). Notably, *TP53*‑mut patients with variant allele frequency (VAF)≥10% showed significantly worse survival than those with VAF<10% (*p = *0.030), whereas VAF<10% cases had survival comparable to *TP53*‑wt. In addition, p53 immunohistochemistry score≥4 yielded an area under the curve (AUC) of 0.79 for *TP53*‐mut prediction. Collectively, *TP53*‐mut in *IDH*‑wt gliomas are prognostically heterogeneous. A VAF≥ 10% identified a potential high‑risk subgroup, with β‐strand mutations associated with the poorest survival in our cohort. p53 immunohistochemistry offers a cost‑effective surrogate screen.

## Graphical abstract

This study reveals that *TP53* mutations in *IDH*‐wildtype gliomas are prognostically heterogeneous. A variant allele frequency (VAF) ≥10% identifies a high‑risk subgroup, and mutations located in the β‑strand region of the DNA‑binding domain confer the poorest outcomes. p53 immunohistochemistry provides a cost‑effective surrogate for mutation detection, enabling refined risk stratification in routine practice.

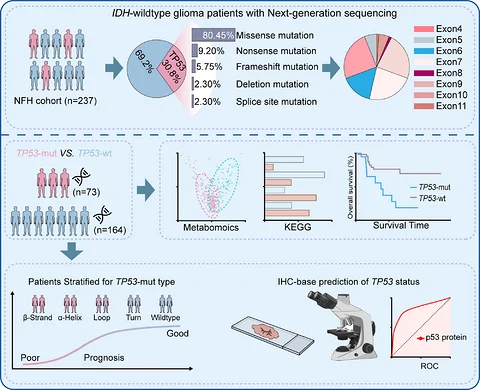

## 1 Introduction

Gliomas are the most common primary brain tumors and are characterized by high morbidity and mortality rates [1]. Despite receiving the standard treatment, the prognosis for glioma patients remains poor, with a five‐year survival rate of less than 10% for those with high‐grade gliomas [2, 3]. Thus, identifying molecular alterations that influence clinical outcomes is crucial for improving diagnosis and therapeutic strategies. Recently, the incorporation of molecular biomarkers, particularly *IDH* mutation (*IDH*‐mut), has fundamentally revised the diagnostic classification of gliomas [4]. In astrocytomas, *TP53* mutation (*TP53*‐mut) serves as an early driver event and is associated with a relatively favorable prognosis, with their clinical significance being well established [5]. In contrast, in *IDH*‐wildtype (*IDH*‐wt) gliomas, the reported *TP53*‐mut frequency varies widely across studies, ranging from 19.4% to 80.0% [6], and can reach 100% in giant cell glioblastoma (GBM) [7]. Currently, its prognostic relevance in *IDH*‐wt gliomas remains unclear.

*TP53* is the most frequently mutated tumor suppressor gene in human cancers to date and is widely recognized as the guardian of the genome [8]. Wildtype p53 is maintained at low levels under physiological conditions but is rapidly stabilized and activated upon genotoxic stress or oncogenic stimuli to transactivate downstream targets and prevent clonal expansion of transformed cells [8]. Large‐scale pan‐cancer sequencing efforts have revealed that *TP53*‐mut is present in approximately 41% of solid tumors, with frequencies varying considerably across cancer types reaching up to 84% in small‐cell lung cancer [9]. Furthermore, data from The Cancer Genome Atlas (TCGA) have demonstrated that *TP53*‐mut not only represents one of the most common genetic alterations, but also significantly associated with genomic instability, complex karyotypes, and increased overall mutational burden [10].

The clinical implications of *TP53*‐mut are not uniform. First, mutation types exhibit functional heterogeneity. Missense mutations, accounting for approximately 74% of cases, not only lead to loss of wildtype p53 tumor suppressor function, but may also exert dominant‐negative effects by interfering with the residual wild‐type allele, or confer novel oncogenic properties, such as promoting epithelial‐mesenchymal transition and metabolic reprogramming [11, 12]. Second, mutational burden has a dose‐dependent effect. Variant allele frequency (VAF) reflects the clonal dominance of mutant alleles within tumors, with high VAF generally indicating truncal mutations rather than subclonal events. In therapy‐related myeloid neoplasms (t‐MN), Shah et al. have suggested that only when *TP53* VAF≥10% does it suffice to define a very high‐risk subgroup, whereas low‐VAF mutations do not affect prognosis [13]. Third, mutation location is of structural biological importance. The vast majority of pathogenic *TP53*‐mut cluster within the DNA‐binding domain (DBD), which is composed of a β‐sandwich, loop regions, and an α‐helix. The β‐strand region is critically important for maintaining the intact spatial conformation and thermodynamic stability of the DBD [14, 15]. Mutations in this region (e.g., V157F, R158H) can lead to p53 protein misfolding, loss of sequence‐specific DNA‐binding ability, and a propensity to form oncogenic amyloid aggregates. By contrast, loop region mutations primarily interfere with the DNA contact interface, and their functional consequences may be relatively limited. Thus, analysis of mutations stratified by secondary structural domains may uncover distinct prognostic values and improve risk stratification.

In astrocytomas, *TP53*‐mut serves as an early driver, co‐occurring with *IDH* and *ATRX* alterations in 70%–80% of cases, and is associated with favorable prognosis [16, 17]. By contrast, in *IDH*‐wt gliomas, *TP53*‐mut arises later, linked to genomic instability, yet their clinical relevance remains controversial [6]. Some studies suggest a poor prognosis, but significance often disappears after *IDH* adjustment [18]. A recent pan‐cancer analysis (*n* > 45,000) linked *TP53*‐mut to worse survival, but this was not robust in the *IDH*‐wt glioma subgroup [19]. These discrepancies have precluded *TP53* from any glioma risk model. Previous glioma studies have overlooked two key issues. First, in *IDH*‐wt gliomas, *TP53* VAF ranges widely (<10%–>80%) with low‐VAF subclonal versus high‐VAF truncal mutations likely having distinct functional impacts—yet no study has tested a VAF threshold for risk stratification. Second, mutations in different p53 secondary structures may drive divergent biology via distinct mechanisms, but no glioma study has mapped mutation sites to the structure for prognostic comparison.

This retrospective study analyzed clinical and next‐generation sequencing (NGS) data from a single‐center cohort of 237 patients with primary *IDH*‐wt glioma. Our aim was to elucidate the prognostic value of *TP53*‐mut in this population and to interrogate the impact of these mutations on OS through the lens of p53 secondary structure, thereby providing a structural perspective to complement existing knowledge. Furthermore, although *TP53*‐mut is a key tumor suppressor event linked to chemotherapy resistance and poor prognosis, conventional sequencing remains costly and inaccessible in many settings. Understanding structural changes can help identify new therapeutic strategies for patients with *TP53*‐mut. Therefore, we also assessed whether immunohistochemistry (IHC) detection of p53 protein expression could serve as a reliable surrogate for predicting *TP53*‐mut status, with the goal of establishing a practical basis for prognostic stratification in primary glioma. The prognostic stratification tool provides clinicians with an actionable framework to identify high‑risk patients early and guide personalized treatment decisions, using p53 IHC as a cost‑effective initial screen to triage patients, particularly in settings where routine NGS is unavailable.

## 2 Results

### 2.1 Baseline Characteristics of Patients With *IDH*‐wt Gliomas in NFH Cohort

After excluding 186 *IDH*‐mut cases from 423 NGS‐profiled primary gliomas, 237 *IDH*‐wt patients were included in this cohort (Figure 1A). Key demographic and clinical characteristics of the cohort are detailed in Table S1. The median age at diagnosis was 48 years (range, 18–78), with a male predominance (male‐to‐female ratio, 1.9:1). The distribution of tumor locations is illustrated in Figure 1B. Cerebral hemispheric involvement predominated (179/237, 75.5%), whereas cerebellar (9/237, 3.8%) involvement was the least frequent. Histopathologically, the cohort comprised 173 GBM and 64 non‐GBM *IDH*‐wt gliomas, including 36 low‑grade and 28 high‑grade cases (Figure S1). Overall, high‐grade gliomas accounted for 84.4% (200/237) of the cases. Regarding initial treatment, surgical resection was performed in 99.2% (235/237) of patients, with pathological biopsies conducted in two cases. Systemic chemotherapy and radiotherapy were administered to 55.3% (131/237) and 53.6% (127/237) of patients, respectively. The median overall survival (OS) for this *IDH*‐wt glioma cohort was 25.8 months.

**FIGURE 1:**
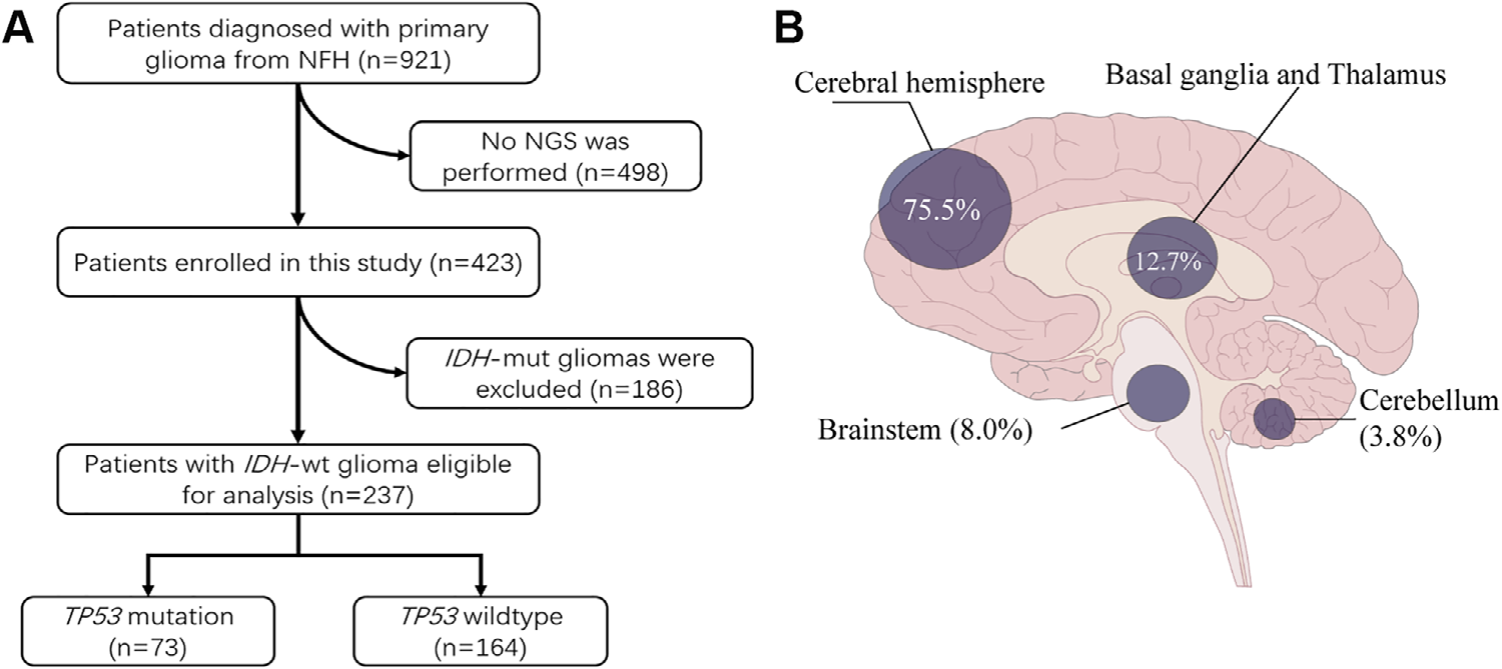
The study flowchart. (A) Flowchart illustrating the selection of the study population in current study. (B) Tumor location at the time of diagnosis in this cohort (*n = *237 tumors).

### 2.2 *TP53*‐Altered Gliomas are Prevalent and Influence Clinical Outcome in *IDH*‐wt Glioma Patients

We further integrated genomic, clinicopathological, and outcome data from the 237 patient NFH cohort and assessed the influence of *TP53*‐mut on patient prognosis. In this cohort, *TP53*‐mut was present in 30.8% (73/237) of cases (Figure 2A). No significant age difference was observed between the *TP53*‐mut (*n = *73) and *TP53*‐wt (*n = *164) subgroups (46.96 ± 16.35 vs. 46.28 ± 15.24, *p = *0.713; Figure 2B). Histologically, microvascular proliferation (MVP) and necrosis were more frequent in the *TP53*‐mut group, but the difference was not statistically significant (Figure 2C). Other baseline characteristics were well‐balanced, as detailed in Table 1. Kaplan–Meier survival analysis demonstrated that patients with *TP53*‐mut had significantly poorer OS compared to those with *TP53*‐wt (HR = 1.99; 95% CI: 1.30–3.05; *p = *0.0004; Figure 2D), with median OS of 18.5 and 36.9 months, respectively. This finding was consistently supported by stratified analyses across various clinicopathological subgroups (Figure 2D). In multivariate Cox regression analysis adjusting for age, tumor category, extent of resection, KPS, chemotherapy, and radiotherapy, *TP53*‐mut remained an independent predictor of worse OS (adjusted HR = 1.91; 95% CI: 1.29–2.84; *p* = 0.001, Figure 2E). Subgroup multivariate analyses in the GBM and non‑GBM cohorts further confirmed *TP53*‐mut as an independent risk factor for OS (GBM: adjusted HR = 1.69, 95% CI: 1.09–2.60, *p = *0.019; non‑GBM: adjusted HR = 3.71, 95% CI: 1.33–10.35, *p* = 0.012, Figures 2E and S2). We further assessed the prognostic relevance of mutation abundance using VAF. The median *TP53*‐mut VAF was 45.6%. Using maximally selected rank statistics, a VAF cut‐off of 10% was identified as the most discriminative threshold within this cohort for stratifying patient outcomes (Figure 2F,G). Only seven patients had a VAF below 10%. Notably, *TP53*‑mut patients with VAF≥10% showed significantly worse survival than those with VAF <10% (HR = 6.57; 95% CI: 2.76–15.67; *p = *0.030), whereas VAF <10% cases had survival comparable to *TP53*‑wt (Figures 2H and S3).

**FIGURE 2:**
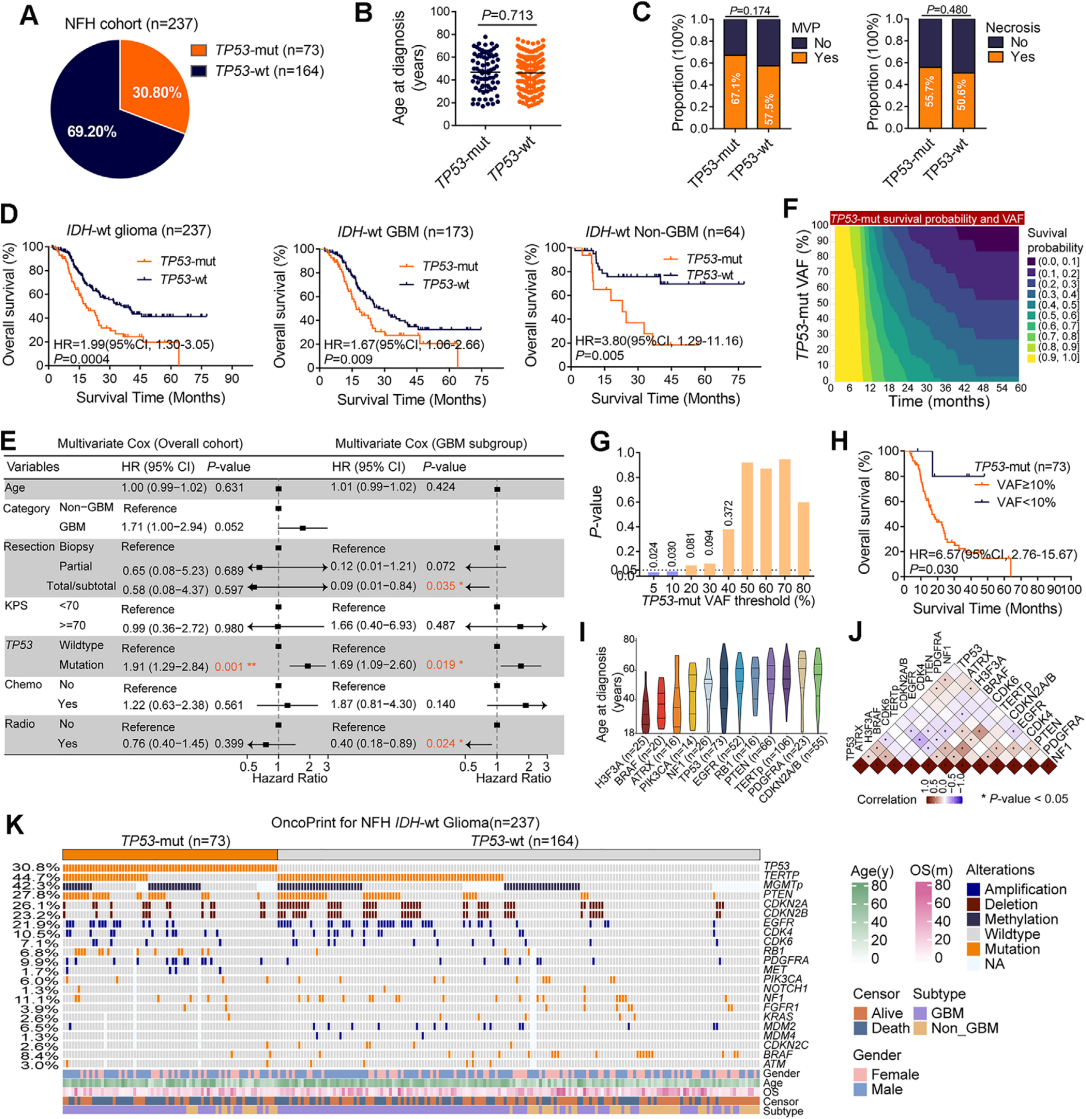
Clinical outcome based on *TP53*‐mut status in 237 *IDH*‐wt glioma patients. (A) Distribution of *TP53*‐mut in NFH cohort. (B) Comparison of ages distribution between *TP53*‐mut (*n = *73) and *TP53*‐wt (*n = *164) subgroups. (C) Comparison of MVP and necrosis rate between the two subgroups. (D) OS based on *TP53*‐mut status. (E) Forest plot multivariate Cox analyses of risk factors related to OS of glioma patients (*n = *237). (F) Survival contour plot visualizing the probability of survival according to *TP53*‐mut VAF. (G) Survival analysis in *IDH*‐wt patients by different cut‐off value of VAF (5%, 10%, 20%, 30%, 40%, 50%, 60%, 70%, and 80%). Survival curve for low and high VAF patient (cut‐off 10%). (H) OS according to *TP53*‐mut VAF cut‐offs. (I) Violin plot showing age at diagnosis separated by molecular alterations in NFH cohort. (J) Heatmap showcasing co‐occurrence and mutually exclusive mutations among differentially mutated genes. (K) Oncoprint heatmap showing distribution and frequency of common molecular alterations.

**TABLE 1:** Comparison of clinicopathological and imaging features between TP53 mutant and wildtype patients in the NFH cohort.

	*TP53* mutation	*TP53* wildtype	*p*‐value
**Patient, *n***	73	164	
**Age at diagnosis**	46.96 ± 16.35	46.28 ± 15.24	0.757
**Gender**			
Male	46	98	0.635
Female	27	66	
**Body mass index**			
BMI<18.5	6	9	0.254
18.5 ≤BMI< 25	44	92	
BMI≥ 25	15	51	
NA	8	12	
**Karnofsky performance status at diagnosis**			
≥ 70	72	156	0.281
< 70	1	8	
**Headache symptom at diagnosis**			
Yes	36	80	0.939
No	37	84	
**Epilepsy at diagnosis**			
Yes	14	40	0.377
No	59	124	
**Tumor location**			
Frontal	14	38	0.888
Parietal	3	9	
Temporal	11	24	
Occipital	0	2	
Cerebellar	2	7	
Brainstem	7	14	
Multifocal growth pattern	36	70	
**The side of tumor**			
Left side	26	66	0.084
Right side	35	73	
Both side	5	11	
Middle	7	14	
**MGMTp status**			
Methylation	28	55	0.588
Unmethylation	34	79	
NA	11	30	
**T1 enhancement**			
Yes	63	130	0.199
No	10	34	
**Extent of resection**			
Biopsy	0	2	0.147
Partial resection	13	16	
Subtotal and total resection	60	146	

Abbreviations: BMI, body mass index; MGMTp, O6‐methylguanine‐DNA methyltransferase promoter; NA, not available.

To characterize the genomic heterogeneity, we analyzed the NGS profiles of all 237 tumors. Age distribution differed by genetic alterations: *EGFR* and *CDKN2A/B* alterations were more common in the elderly, while *H3F3A* and *BRAF* mutations clustered in younger patients (Figure 2I). Assessment of co‐mutated genes showed that *TP53*‐mut frequently co‐occurred with *TERT*p (29/237, 12.2%), *PDGFRA* (13/237, 5.5%), and *PTEN* (27/237, 11.4%) alterations, but were mutually exclusive with *BRAF* mutation (Figure 2J). An oncoprint summary of the altered genes highlighted distinct mutational signatures between *TP53*‐mut and *TP53*‐wt tumors, with the former exhibiting higher co‐alteration burdens (Figure 2K). *TP53*‐mut predominated in GBM (56/73, 76.7%) and were confined to WHO Grade 3–4 non‑GBM tumors (13/73, 17.8%). Co‑alteration analysis revealed frequent co‑occurrence with *PDGFRA* and *PTEN*, and mutual exclusivity with *BRAF* (235/237, 99.2%). Focal *MDM2/MDM4* amplifications were detected in some GBM lacking *TP53* or *CDKN2A/B* alterations, and core cell‑cycle regulators (*CDKN2A/B*, *CDK4/6*, *RB1*, and *CDKN2C*) showed minimal overlap, consistent with known mutual exclusivity patterns in *IDH*‑wt glioma pathways.

### 2.3 Clinical Features and Molecular Alteration Vary Across *IDH*‐wt Glioma Subtypes

We further stratified *IDH*‐wt gliomas into four subtypes: GBM/*TP53*‐wt, GBM/*TP53*‐mut, non‐GBM/*TP53*‐wt, and non‐GBM/*TP53*‐mut. The clinical characteristics, tumor location, molecular feature, and outcome of four subtypes are summarized in Figure 3A. Analysis of age distribution revealed that GBM/*TP53*‐wt patients had the highest median age at diagnosis (53 years), followed by GBM/*TP53*‐mut (51 years), non‐GBM/*TP53*‐wt (48 years), and non‐GBM/*TP53*‐mut patients (34 years; Figure 3A). The GBM/*TP53*‐wt subtype was the most prevalent, constituting 49.4% (117/237) of the cohort. Beyond differences in age and tumor location, molecular profiles also varied substantially among subtypes (Figure 3A). In terms of prognosis, patients with non‐GBM/*TP53*‐wt tumors exhibited the most favorable outcomes, while those with GBM/*TP53*‐mut had the poorest survival, with a median OS of only 16 months. Evaluation of four core tumorigenic pathways—RTK‐driven, RAS/MAPK‐driven, histone‐driven, and cell cycle‐driven—showed that the GBM/*TP53*‐mut subtype displayed the highest median number of pathway co‐alterations (median = 2, Figure 3B). A total of 51.0% of GBM/*TP53*‐mut patients harbored ≥ 2 pathway alterations, predominantly RTKs (44.6%) and cell cycle (62.5%), with 35.7% of cases showing co‑occurrence of both. In contrast, non‐GBM/*TP53*‐wt cases demonstrated frequent RAS/MAPK (40.4%) but low cell cycle pathway involvement (8.5%), whereas non‐GBM/*TP53*‐mut cases showed minimal activation across both pathways (6.4%).

**FIGURE 3:**
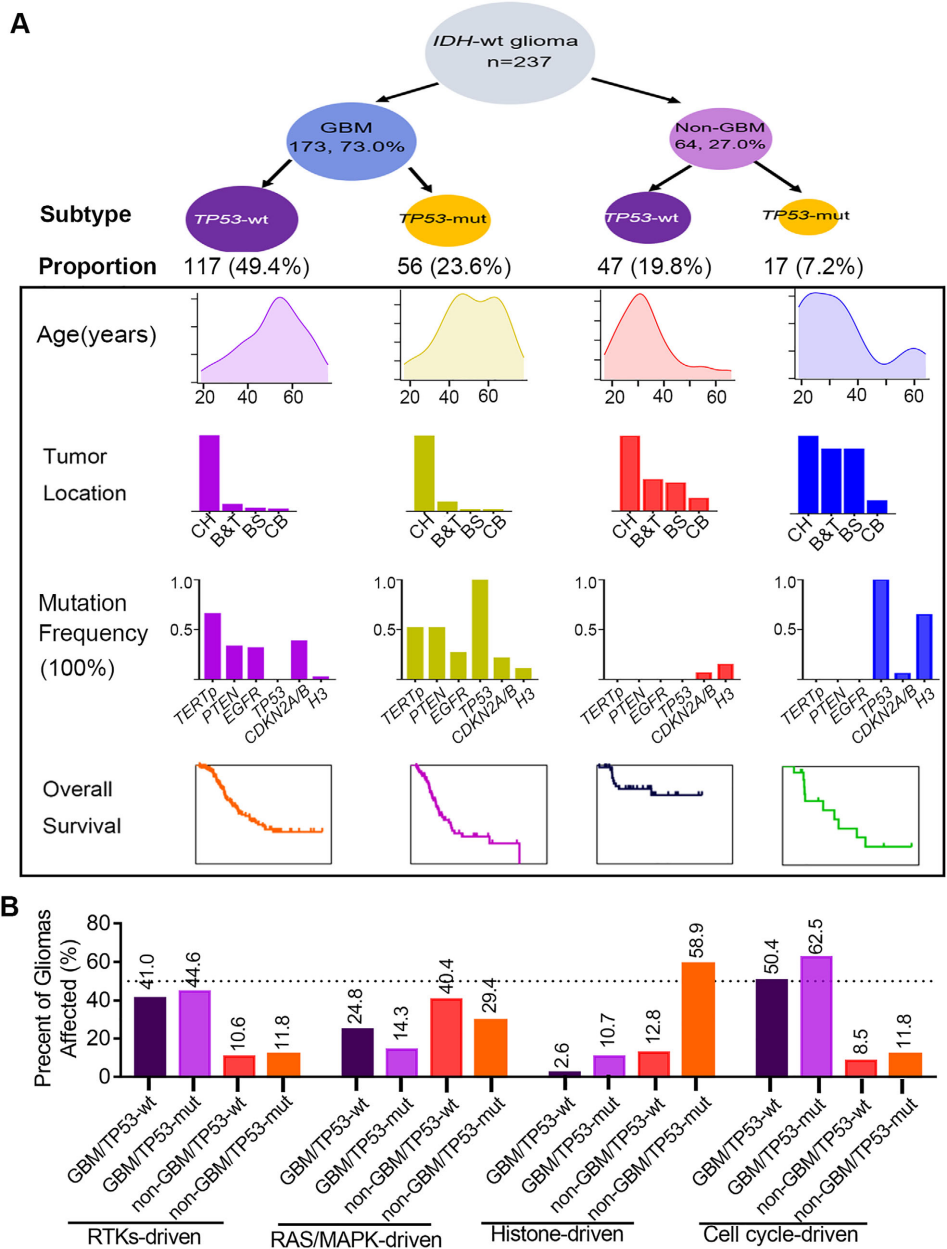
NFH Cohort overview (*n = *237). (A) Summary of the pooled *IDH*‐wt glioma cohort. (B) Proportion of glioma subgroups with an affected tumorigenic pathway: RTK‐driven, RAS/MAPK‐driven, Histone‐driven, and cell cycle‐driven.

### 2.4 *TP53*‐mut in the β‐Strand Region was Associated With Inferior Clinical Outcome

To determine whether the site of mutation confers differential prognostic value, we mapped the *TP53* alterations to their corresponding exonic domains. The most commonly affected exons were Exon 5 (24.39%), Exons 8 and 7 (each 23.17%), Exon 6 (15.85%), and other exons (Figure 4A). Missense mutations constituted the predominant type (59/73, 80.45%; Figure 4B). Details of *TP53*‐mut were shown in Figure 4C. The vast majority (62/73, 85%) of mutations were located within the DBD, with codons R248 (*n = *8) and R273 (*n = *11) being the most frequently affected. Mutations in the *TP53* DBD are predominantly missense mutations (Figure S4).

**FIGURE 4:**
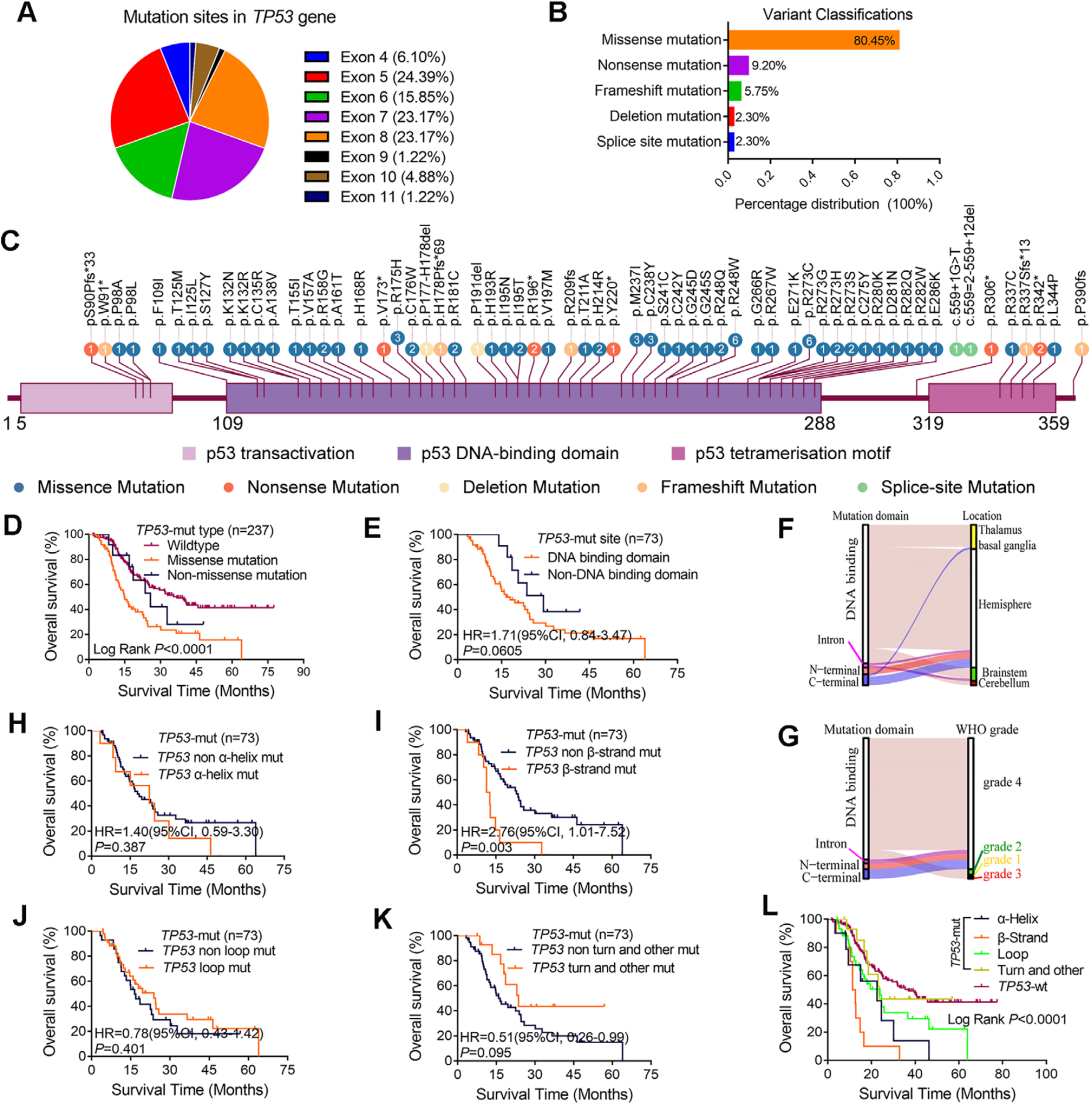
Proportions of different mutation types of *TP53*, and prognostic stratification of *TP53* alterations in *IDH*‐wt gliomas (*n = *237). (A) Distribution proportion of *TP53*‐mut in different exons. (B) Proportions of different mutation types of *TP53*. (C) Lollipop plot demonstrating all *TP53* variants in this cohort. The number of times the variant observed in this current cohort was displayed within the lollipops. (D) Kaplan–Meier curves comparing OS among three groups: *TP53* missense mutation, *TP53* non‐missense mutation, and *TP53*‐wt. (E) Kaplan–Meier curves comparing OS between patients with *TP53*‐mut located within the DBD versus those outside the DBD. (F‐G) *TP53*‐mut domains in relation to tumor location and WHO grade. (H‐L) Kaplan–Meier survival curves for each secondary structural category: α‑helix (H), β‑strand (I), loop (J), turn/other regions (K), and the combined *TP53*‐wt reference group (L).

We next evaluated whether mutation type and functional domain affected prognosis. Missense mutation, as the predominant type, was associated with the most significant effect on survival (*p *< 0.0001, Figure 4D). Patients with *TP53* missense mutations, non‐missense mutations, and *TP53*‐wt had median OS of 15.0, 25.8, and 36.9 months, respectively. We next compared outcomes between patients with DBD and non‑DBD mutations. Although DBD mutations showed a trend toward poorer OS, the difference did not reach statistical significance (*p = *0.0605, Figure 4E). No significant associations were found between mutational domains and tumor location or grade (Figures 4F,G). As for the perspective of the structural subgroup, *TP53*‐mut was classified according to the secondary structure of the p53 protein, including mutations in the α‐helix, β‐strand, loop, turn, and other regions. By secondary structure, mutation frequencies were: α‑helix 13.7%, β‑strand 13.7%, loop 57.4%, and turn/other 15.2%. Median OS was 22.5 months for α‑helix, 12.0 months for β‑strand, 23.9 months for loop, and 23.6 months for turn/other region mutations. Notably, β‑strand mutations, which were identified in 10 patients (13.7% of all *TP53*‑mut cases), conferred the worst prognosis among all *TP53*‑mut subgroups and versus *TP53*‑wt (Figures 4H,L).

### 2.5 Integrative Analysis of Transcriptomic and Metabolomic Profiles in *TP53*‐wt Versus *TP53*‐mut Gliomas and Drug Sensitivity Assessment in Organoid Models

To further explore the functional impact of *TP53*‐mut, we performed integrated transcriptomic (*n = *6) and metabolomic (*n = *6) profiling on GBM tumor tissues (Figure 5A). KEGG enrichment analysis revealed that differentially expressed genes were significantly enriched in pathways related to core oncogenic signaling (PI3K‐Akt, Ras, and TGF‐beta), metabolic reprogramming (central carbon metabolism, glycolysis), tumor microenvironment remodeling (HIF‐1, efferocytosis), and neurodevelopmental/ synaptic signaling (axon guidance, glutamatergic synapse) (Figure 5B). Figure 5C further delineated distinct transcriptional programs between *TP53*‐wt and *TP53*‐mut subgroups: the latter showed upregulation of genes involved in Ras signaling pathway, TGF‐beta signaling pathway, Hippo signaling pathway, and the PI3K/AKT pathway. Metabolomic profiling identified 255 significantly differential metabolites in *TP53*‐mut subgroup relative to *TP53*‐wt controls, which mainly enriched in “alanine, aspartate, and glutamate metabolism” and “D‐Amino acid metabolism” terms (Figure 5D). Further investigation indicated that these differentially expressed metabolites were primarily linked to activation of lipid and amino acid metabolism (Figure 5E).

**FIGURE 5:**
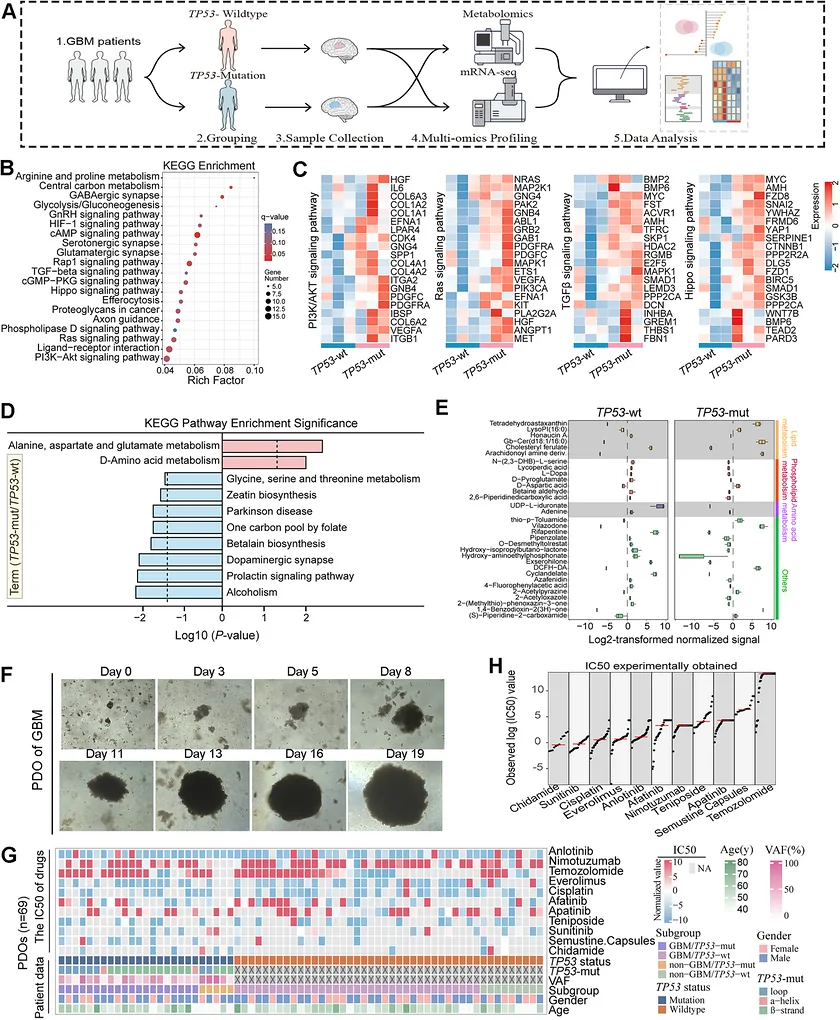
Integrative analysis of molecular signatures using transcriptomic and metabolomic profiles. (A) Schematic of tumor tissues collection and analysis of data. (B) KEGG enrichment analysis for mRNA‐seq data between *TP53*‐mut and *TP53*‐wt tissues (*n = *6). (C) Heatmap of differentially expressed genes in various KEGG term. (D) Metabolic analyses revealed the number of up‐ and down‐regulated differential metabolites (*n = *6). (E) Metabolomic analysis revealed significantly differential metabolites between the *TP53*‐mut and *TP53*‐wt subgroups, along with their associated metabolic pathways. (F) Schematic of the PDOs culture timeline. (G) The correlation heatmap between the IC50 values of anti‐tumor drugs and *TP53*‐mut features. (H) The half‐maximal inhibitory concentrations (IC50) of multiple drugs were determined in *IDH*‐wt glioma organoids.

To evaluate the influence of *TP53* status on therapeutic response, we established patient‐derived organoids (PDOs) from primary *IDH*‐wt gliomas (Figure 5F). Across 69 PDOs, IC50 values were determined for a panel of anticancer agents. Summary data linking *TP53*‐mut status to drug sensitivity are presented in Figure 5G. Overall, *IDH*‐wt glioma organoids displayed relative resistance to temozolomide. *TP53*‐mut PDOs tended to show greater resistance to temozolomide and suggested higher sensitivity to everolimus, whereas they remained broadly sensitive to chidamide and sunitinib (Figure 5H).

### 2.6 Correlation Between p53 Expression and *TP53*‐mut in 237 *IDH*‐wt Gliomas

IHC is a commonly used tool by pathologists. To determine whether p53 protein IHC score could serve as a predictive marker for *TP53*‐mut status, we performed p53 IHC staining on tumor tissue from 237 glioma patients. Five staining patterns were identified (Figure 6A): focal weak positivity, patchy heterogeneous, complete absence, diffuse strong nuclear positivity, and overexpression with cytoplasmic retention. We next assessed the association of *TP53*‑mut status with p53 IHC staining. In the *TP53*‑wt group, staining was predominantly focal weak (65%) and patchy heterogeneous (30%), whereas in the *TP53*‑mut group, diffuse strong nuclear positivity dominated (64%) (Figure 6B). Figure 6C presents the visualization of WHO grades, p53 IHC scores, *TP53*‐mut types, and *TP53*‐mut VAF data obtained from 237 patients. The p53 IHC score was significantly higher in *TP53*‐mut tumors than in *TP53*‐wt tumors (*p *< 0.0001), with the highest scores observed in the missense mutation subgroup relative to other mutation types (*p *< 0.0001; Figure 6D). Similarly, Western blot analysis showed that p53 expression was increased in *TP53*‐mut tissues (Figure 6E).

**FIGURE 6:**
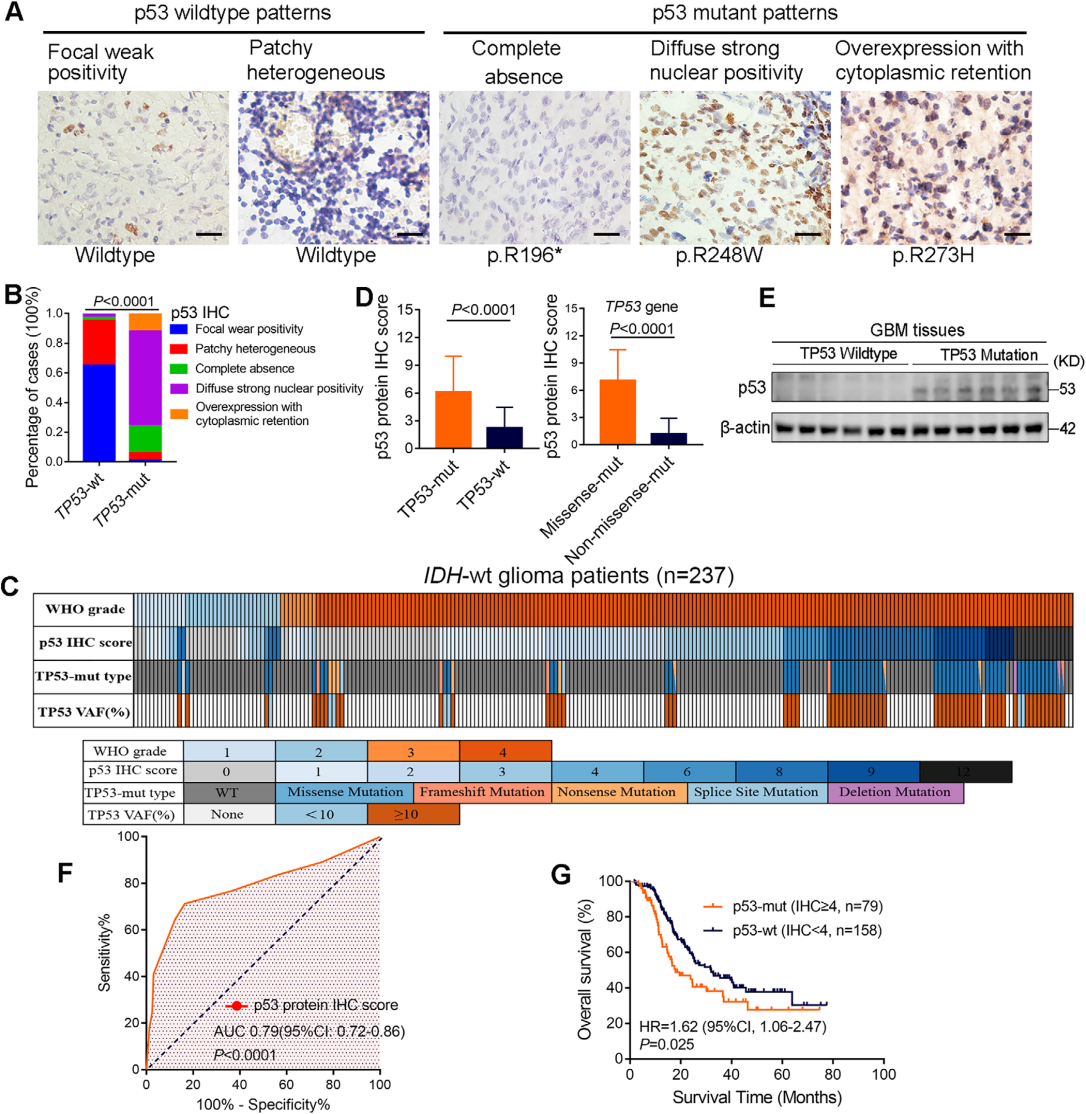
p53 protein expression predicts *TP53* status. (A) Examples of p53 wildtype (focal weak positivity and patchy heterogeneous), p53 mutation staining (complete absence, diffuse strong nuclear positivity and overexpression with cytoplasmic retention). Scale bar represents 50 µm. (B) Concordance on the basis or presence of *TP53*‐mut with p53 protein staining intensity. (C) Visualization of the relationship between WHO grade, p53 IHC score and the type/VAF of *TP53*‐mut in NFH cohort. Each row corresponds to one sample, and each column corresponds to one feature. (D) Comparison of p53 IHC scores between *TP53*‐mut (*n = *73) and *TP53*‐wt (*n = *164) tumors. (E) p53 expression in the primary GBM tissues with *TP53*‐wt and *TP53*‐mut by Western blot. (F) ROC curve for prediction of *TP53*‐mut by p53 protein IHC score in glioma patients (*n = *237). (G) OS of patients with *IDH*‐wt glioma, stratified by p53 status.

Receiver operating characteristic (ROC) curve analysis assessed the predictive performance of the p53 IHC score for *TP53*‐mut status, yielding an area under the curve (AUC) of 0.79 (95% CI: 0.72–0.86, *p *< 0.0001; Figure 6F). At the Youden‐index‐derived cut‐off of 4.0, sensitivity was 71.23% (52/73), specificity 83.54% (137/164), with a positive predictive value (PPV) of 65.82% (52/79) and negative predictive value (NPV) of 86.71% (137/158). Using this threshold (IHC score ≥ 4) to define p53‐mut, patients in the p53‐mut group (*n = *79) had significantly worse OS than those with IHC < 4 (*n = *158; *p = *0.02; Figure 6G).

## 3 Discussion

*TP53* is frequently mutated in cancers, yet increasing evidence indicates that different mutation subtypes exert distinct effects on p53 function, tumor progression, and patient survival [20]. Thus, refined subclassification by mutation type and functional impairment is essential for better prognostic stratification. In a retrospective cohort of 237 primary *IDH*‑wt glioma patients, we found *TP53*‐mut in 30.8% of cases, significantly associated with reduced OS. Importantly, we establish that not all *TP53*‐mut confer equivalent risk; rather, prognosis is modulated by both the VAF and the specific structural domain affected. Specifically, a VAF≥ 10% was associated with worse outcomes, and mutations within the β‑strand region of the DNA‑binding domain showed the shortest observed survival; however, both findings are exploratory and require independent validation. Finally, we validated p53 immunohistochemistry as a reliable and cost‑effective surrogate for *TP53*‐mut status, supporting its routine incorporation into pathological risk stratification.

The frequency of *TP53*‐mut observed in our cohort (30.8%) aligns with prior reports in *IDH*‐wt gliomas, which generally range from 30% to 50% [6, 21]. This stands in contrast to the higher prevalence (up to 80%) typically seen in *IDH*‐mut astrocytomas, where *TP53* alteration often occurs as an early initiating event. Murnyak and Huang reported that *TP53*‐mut is a significant independent predictor of poorer OS in patients with lower‐grade glioma in 45,000 sample cohort [19]. This association holds true across various molecular and histological subgroups, including *IDH*‐mut astrocytomas and oligodendrogliomas, underscoring the critical prognostic value of *TP53* status irrespective of other classifications. However, a study by Schmidt et al. involving 97 unselected GBM patients, found that *TP53*‐mut was a significant favorable prognostic factor, with mutation carriers showing a significantly longer median survival compared to wildtype patients [22]. This association remained significant even after adjusting for patient age in a multivariate Cox regression analysis and was observed independently in the subgroup of primary GBM. This study's primary limitation is its relatively small sample size, particularly including secondary GBM [22]. In addition, Di Nunno et al. reported that *TP53*‐mut status was not significantly associated with OS in the *IDH*‐wt GBM patients [6]. This discrepancy may be attributed to differences in cohort selection, the timing of *TP53* alteration during tumor evolution, and the co‐occurrence of other genetic events. A key finding of our study is the prognostic significance of *TP53* VAF. Our exploratory analysis identified a VAF of 10% as a potential prognostic threshold, with higher mutational burden associated with significantly worse survival. This observation resonates with findings in other malignancies, such as myeloid neoplasms, where a VAF≥ 10% delineates a high‐risk subgroup [23]. Biologically, a higher VAF likely reflects clonal dominance of the *TP53*‐mut population, suggesting a more fundamental role in tumor maintenance and progression [24, 25]. The quantitative assessment of VAF thus adds a layer of prognostic refinement beyond mere mutational presence, potentially informing more personalized surveillance and therapeutic strategies.

Beyond mutation dosage, the structural context of the mutation emerges as a critical determinant of outcome. The main forms of protein secondary structure include α‐helix, β‐strand, loop, and turn [26]. Mutations in the *TP53* gene may lead to alterations in the protein's secondary structure, thereby affecting its function and stability [27, 28]. The vast majority (86.3%) of mutations in our cohort resided within the DBD, with classic hotspot residues R248 and R273 being most frequently affected. Although mutations in DBD showed a trend toward poorer survival compared to non‑DBD mutations, the difference did not reach statistical significance. We further explore the impact of mutations related to the secondary structure of protein on clinical outcomes, including α‐helix, β‐strand, loop, turn and others. We found that *TP53*‐mut in the β‐strand region had an exceptionally poor outcome. The p53 β‐sandwich is key to the stability of the DNA‐binding surface [14]. Mutations in this region can induce profound conformational changes, abrogating DNA binding and transactivation while potentially promoting aggressive gain‐of‐function phenotypes through altered protein‐protein interactions or stability [29]. Mechanistically, β‐strand mutations disrupt the β‐sandwich core via altered hydrogen bonds and hydrophobic packing, reducing thermodynamic stability and allosterically impairing DNA binding. The resulting misfolded p53 exposes hydrophobic patches, promoting amyloid‐like oligomerization and gain‐of‐function interactions with HSF‐1 and p63 [30, 31]. These cooperative effects drive tumor aggressiveness and chemoresistance, explaining the poor prognosis of *IDH*‐wt gliomas with β‐strand mutations. Zhang et al. found that *TP53*‐mut located in the α‐helix region is associated with significantly worse OS and progression‐free survival in advanced EGFR‐mutant NSCLC patients treated with third‐generation EGFR‐TKIs [32]. This specific structural mutation serves as an independent negative predictive factor for poorer clinical outcomes. Understanding structural changes can help identify new therapeutic strategies for patients with *TP53*‐mut. Mutations at the R248 and R273 sites of the *TP53* gene are classified as DNA contact surface mutations [33]. They impair the ability of the p53 protein to bind to DNA, leading to a loss of transcriptional activity and tumor‐suppressor function, and potentially conferring cancer‐promoting properties. Yu Zhang et al. found that elucidating how the loss of p53 function promotes tumor progression through the derepression of PCSK9 transcriptional inhibition provides a precision therapeutic target for p53‐mut anaplastic thyroid cancer [34].

The detection of *TP53* genomic alterations using NGS requires a demanding laboratory setup, is time‐consuming and costly, thereby limiting its broad application. Consequently, there is a pressing need for a simplified and more economical alternative to NGS‐based testing. In this study, we found that p53 IHC served as a cost‐effective predictive indicator for *TP53*‐mut, offering a practical tool for risk stratification in clinical practice. Jin et al. reported that IHC detection of p53 expression effectively predicts *TP53*‐mut with an accuracy of 82.1% in diffuse large B‐cell lymphoma [35]. While IHC cannot distinguish between different mutation types or structural domains, its cost‐effectiveness and widespread availability make it an invaluable first‐line test for identifying patients likely to harbor clinically significant *TP53* alterations, particularly in resource‐limited settings where routine NGS is not feasible. While this predictive effect possesses clinical utility, it is not yet robust enough to be considered fully reliable.

Several limitations should be acknowledged. First, all subgroup analyses—including the evaluation of VAF thresholds and secondary structural domains—were exploratory; therefore, these findings are hypothesis‑generating and require validation in large, prospective, multi‑center cohorts. Second, small subgroup sizes (e.g., β‐strand mutations) reduce statistical power; these exploratory findings require validation in larger cohorts. Third, the *TP53* VAF ≥10% threshold was derived from exploratory analysis and carries data‐driven bias risk, necessitating independent verification. Fourth, transcriptomic, metabolomic, and organoid drug‐response analyses were based on limited samples; their results are hypothesis‐generating and cannot exclude confounding by co‐occurring alterations. Fifth, p53 IHC shows only moderate discriminatory performance and is particularly insensitive to truncating mutations; it should serve as a pre‐screening tool rather than a replacement for NGS in critical cases. Sixth, the high missing rate of *MGMT*p methylation status (17.3%, 41/237) precluded its inclusion in the multivariable Cox regression model. Seventh, the PDO drug–response analysis had limited samples and small subgroups. Thus, the findings should be viewed as descriptive and hypothesis‑generating, not as definitive evidence of a *TP53*‑specific effect. Future research should investigate the distinct biological mechanisms driven by mutations in specific structural domains, especially the β‑strand, which may reveal novel therapeutic vulnerabilities for this glioma subgroup.

## 4 Materials and Methods

### 4.1 Patient Cohort

This study enrolled patients diagnosed with primary glioma at Nanfang Hospital from January 2019 to April 2024, with available clinical NGS data. Molecular findings and electronic medical records were retrospectively reviewed manually. Patients harboring *IDH*‐mut were excluded. Ultimately, 237 distinct patients met the inclusion criteria with adequate data for analysis. Follow‑up was updated through October 2025, with a median follow‑up duration calculated from the date of diagnosis.

### 4.2 Clinical and Neuroimaging Data Acquisition

The following clinical parameters were recorded for each patient: age, sex, presenting symptomatology, extent of surgical resection, histopathological grade, and follow‑up status. Preoperative MRI was performed on a 3.0T Siemens Skyra scanner with a 20‑channel head coil. The protocol included axial T1‑weighted (pre‑ and post‑contrast), T2‑weighted, and fluid‑attenuated inversion recovery (FLAIR) sequences. Acquisition parameters were: TR = 2000 ms, TE = 9 ms, TI = 2500 ms (FLAIR), flip angle = 90°, FOV = 230 × 187 mm, matrix = 256 × 256 (in‑plane resolution ∼0.9 × 0.7 mm), slice thickness = 6 mm, and inter‑slice gap = 1.2 mm. Sequence selection followed consensus recommendations for neuro–oncologic imaging. All images were retrieved from picture archiving and communication system (PACS). Two neurosurgeons blinded to clinical and pathological data independently reviewed all MRI scans. Discrepancies were resolved by discussion or, if needed, adjudication by a senior neurosurgeon. These MRI data were primarily used to determine tumor anatomical location and to provide radiological reference for histopathological assessment.

### 4.3 Sample Processing and Clinico‐Genomic Sequencing

Formalin‐fixed paraffin‐embedded tumor slides or frozen fresh tumor tissues were collected along with paired blood samples. The sequencing was performed at the Simcere Diagnostics Laboratory (SimcereDx, Nanjing, China). Genomic DNA (gDNA) from tumor tissues was extracted using the Tissue Sample DNA Extraction Kit (Kai Shuo), while gDNA from paired leukocytes was extracted using MagMAX DNA Multi‐Sample Ultra Kit (Thermo). The concentration of the extracted gDNA was quantified with the Qubit dsDNA HS Assay Kit using a Qubit fluorometer (Thermo Fisher Scientific), and its quality was assessed using the Agilent 4200 TapeStation System. Next, 200 ng of the qualified gDNA was enzymatically fragmented into 200 to 300 bp pieces. The ends of these gDNA fragments were repaired with the VAHTS Universal Plus DNA Library Prep Kit (Vazyme Biotech, Nanjing, China; Catalog No. ND617). Target enrichment was conducted using the hybrid‐capture‐based Neuro Onco 360 panel (SimcereDx) with the KAPA HyperCapture Reagent Kit (Roche, USA) and associated probes. The qualified DNA libraries were then subjected to paired‐end sequencing on an Illumina NovaSeq 6000 platform (Illumina, San Diego, CA, USA), generating 150‐bp reads. The sequencing data for each sample were stored in FASTQ format. The fastp software (version 2.20.0) was used for quality control, adapter trimming, and removal of low‐quality bases. The paired‐end reads were aligned to the GRCh37 (hg19) reference genome using the Burrows–Wheeler Aligner algorithm (BWA‐MEM version 0.7.17).

*TP53*‐mut was also classified according to their location in secondary protein structures (i.e., α‐helix, β‐strand, loop, turn) with reference to the p53 mutation database (http://p53.fr/tp53‐database/mutation‐database) and UniProt (https://www.uniprot.org/).

### 4.4 IHC Assay

We evaluated p53 expression in tumor tissues via IHC [36]. Briefly, 4‐µm sections from paraffin‐embedded blocks were deparaffinized and rehydrated. Antigen retrieval was performed by pressure‐cooking in citrate buffer (pH 6.0) for 5 min, followed by endogenous peroxidase blockade with 0.3% H_2_O_2_. After blocking with 5% BSA for 1 h, the sections were incubated with a primary anti‐p53 antibody (Cat. no. ZM‐0408; Zhongshan Golden Bridge Biotechnology) and subsequently with an HRP‐conjugated secondary antibody (Cat. no. SAP‐9100; same supplier). Signal visualization was achieved using diaminobenzidine (DAB), and the sections were counterstained with hematoxylin prior to microscopic examination. In accordance with a previously established methodology [37], p53 immunoreactivity was evaluated using an immunoreactivity score (IRS), calculated as the product of the staining intensity and the percentage of positive cells.

### 4.5 RNA‐seq Analysis

Tumor specimens preserved in liquid nitrogen were subjected to RNA sequencing for comparison between *TP53*‐wt and *TP53*‐mut groups. Total RNA was extracted using Trizol reagent (Invitrogen, USA). RNA concentration and purity were measured with a Nanodrop 8000 spectrophotometer (A260/280 ratio maintained at 1.8–2.0), and integrity was assessed using an Agilent 2100 Bioanalyzer (Agilent Technologies, USA). Only RNA samples meeting the following quality criteria were used for subsequent analysis: RNA Integrity Number (RIN) ≥ 7.0 and 28S:18S rRNA ratio ≥ 1.5. RNA‐seq libraries were constructed by BGI Genomics (Shenzhen, China). Briefly, ribosomal RNA was removed using the RNaseH method (Epicentre Technologies, USA), and polyA‐tailed mRNA was enriched with the Ribo–Zero Magnetic Gold Kit (Illumina, USA). The purified RNA was fragmented and reverse‐transcribed into first‐strand cDNA using random hexamer primers, followed by second‐strand synthesis to generate double‐stranded cDNA. After end repair, 5’‐phosphorylation, and A‐tailing, adapters were ligated to the cDNA fragments. The libraries were amplified and enriched by qRT‐PCR, quality‐checked on an Agilent 2100 system, and quantified with the KAPA Library Quantification Kit (KAPA Biosystems, South Africa). Finally, sequencing was performed on the BGISEQ‐500 platform.

### 4.6 Metabolomics Analysis

The metabolomics analysis work was completed by Shenzhen Huada Gene Technology Co. Ltd. (Shenzhen, China).

### 4.7 GBM Organoid Construction and Culture

Following surgical resection, tumor tissue was immediately suspended in an ice‐cold culture medium and transported to the laboratory on ice, with all procedures completed within 30 min. GBM organoids were subsequently generated using a previously described protocol [36]. The organoids were plated in six‐well, ultra‐low attachment plates (Corning; 3471) and cultured in a humidified incubator at 37°C, 5% CO_2_, and 21% oxygen under constant agitation at 120 rpm. The culture medium of GBM organoid was replaced every 48 h.

### 4.8 Statistical Analysis

Baseline characteristics were summarized using descriptive statistics. Categorical and continuous variables were analyzed by the Chi‐squared test and the independent samples *t*‐test (or Mann–Whitney *U* test), respectively. Survival distributions were generated with the Kaplan–Meier method and compared using the log‐rank test. The discriminatory accuracy of the IHC score in predicting *TP53*‐mut status was evaluated by ROC curve analysis. A two‐sided *p*‐value < 0.05 was considered statistically significant for all tests. All analyses were performed using SPSS (version 27.0), R (version 4.3.1), and GraphPad Prism (version 8.0).

## Author Contributions

SQ.Q. and GL.H. conceptualized the study, created the methodology, and wrote the original draft. Z.Y., KL.L., YD.H., C.X., QM.P. and HR.L. performed the data collection. JX.W., GZ.Y., ZY.L. and GL.L. conducted the prognosis follow‐up of patients and the IHC assay. C.C. and YO.Q. performed the statistical analysis. ZY.C., X.Z. and ST.Q. supervised the project. SQ.Q. performed the draft revision. All the authors have read and approved the final manuscript.

## Funding

This work was supported by National Natural Science Foundation of China (82473161), GuangDong Basic and Applied Basic Research Foundation (2023A1515111129), Beijing Medical and Health Foundation (YWJKJJHKYJJ‐ABS‐2025‐024), and Southern Medical University Undergraduate Innovation and Entrepreneurship Training Program (12121024).

## Ethics Statement

This study was approved by Nanfang Hospital of Southern Medical University Institutional Review Board (No. NFEC‐2025‐537) and adhered to the tenets of the Declaration of Helsinki.

## Conflicts of Interest

The authors declare no conflicts of interest.

## Supporting information




**Supporting file 1**: mco271041‐sup‐0001‐SuppMat.docx

## Data Availability

The raw sequencing data reported in this paper have been deposited in the Genome Sequence Archive (GSA‐Human) at the National Genomics Data Center, China National Center for Bioinformation / Beijing Institute of Genomics, Chinese Academy of Sciences (https://ngdc.cncb.ac.cn/gsa‐human). The genomic DNA sequencing data (DNAseq) are accessible under accession number HRA019845, and the transcriptomic sequencing data (RNAseq) are accessible under accession number HRA020379. The metabolomics data have been deposited in the OMIX database (https://ngdc.cncb.ac.cn/omix) under accession number OMIX019658. Data access requests can be submitted through the CNCB‐NGDC portal and require approval from the designated Data Access Committee. Requests may also be directed to the corresponding author (qushq3@163.com), subject to institutional review board approval and execution of a data‐sharing agreement.
