# Supplementary material for: TP53 Mutation Heterogeneity Refines Prognostic Stratification in IDH‐Wildtype Glioma
Source: MedComm (2020). 2026 Oct 4;7(10):e71041. doi: 10.1002/mco2.71041 (PMC13635792; doi:10.1002/mco2.71041)
Supplement: Supplementary file 1 [file MCO2-7-e71041-s001.docx]

**Supplementary Information**

**Title: *TP53* Mutation Heterogeneity Refines Prognostic Stratification in *IDH*-Wildtype Glioma**

Kuilin Liao^1,2,3,4,5^, Zhi Ye^1,2,3,4^, Songtao Qi ^1,2,3,4^, Chang Xu^1,2,3,4^, Yudi Huang^4,5^, Zhiyong Li^1,2,3,4^,Guozhong Yi^1,2,3,4^, Yuou Qin^4,5^,Qiuming Pan^1,2,3,4^,Guiling Liu^1^, Chao Chen^1,2,3,4^, Zhuoyang Chen^1,2,3,4^, Hongrui Li ^4,5^, Junxi Wang^4,5^, Xi’an Zhang^1,2,3,4^, Guanglong Huang^1,2,3,4*^, Shanqiang Qu^1,2,3,4,6*^

^1^ Department of Neurosurgery, Nanfang Hospital, Southern Medical University, Guangzhou, Guangdong 510515, the People's Republic of China

^2^ Nanfang Glioma Center, Nanfang Hospital, Southern Medical University, Guangzhou, Guangdong 510515, the People's Republic of China

^3^ Institute of Brain disease, Nanfang Hospital, Southern Medical University, Guangzhou, Guangdong 510515, the People's Republic of China

^4^ Laboratory for Precision Neurosurgery, Nanfang Hospital, Southern Medical University, Guangzhou, Guangdong 510515, the People's Republic of China

^5^ The First Clinical School of Medicine, Southern Medical University, Guangzhou, Guangdong 510515, the People's Republic of China

^6^ Department of Neurosurgery, Ganzhou Hospital-Nanfang Hospital, Southern Medical University, Ganzhou, Jiangxi 341000, the People's Republic of China

Kuilin Liao, Zhi Ye and Songtao Qi contributed equally to this work.

Running title: *TP53*‑mut in IDH‑wt Glioma.

***Correspondence**

Shanqiang Qu, M.D., Ph.D.,

Department of Neurosurgery, Nanfang Hospital, Southern Medical University,

Guangzhou Dadao Bei Street 1838#, Guangzhou 510515, the People's Republic of China.

Tel: 86-20-62787231,

Fax: 86-20-62787231,

E-mail: qushq3@163.com

ORCID: 0000-0002-0101-2709

Guanglong Huang, M.D., Ph.D.,

Department of Neurosurgery, Nanfang Hospital, Southern Medical University,

Guangzhou Dadao Bei Street 1838#, Guangzhou 510515, the People's Republic of China.

Tel: 86-20-62787230,

Fax: 86-20-62787230,

E-mail: hgl1020@163.com

ORCID: 0000-0002-1589-9202

**Materials Methods**

**Diagnostic Criteria for Non-GBM *IDH*-wt Gliomas**

All tumors were histologically classified according to the 2021 WHO Classification of Tumors of the Central Nervous System (WHO CNS5). *IDH* mutation status was determined by targeted sequencing of *IDH1* and *IDH2*. In *IDH*-wildtype cases, glioblastoma (GBM) was diagnosed when tumors exhibited histological features of grade 4 astrocytoma (i.e., microvascular proliferation, necrosis, or both), or met the molecular diagnostic criteria for GBM, including *TERT* promoter mutation, *EGFR* amplification, and/or combined whole-chromosome 7 gain and chromosome 10 loss. *IDH*-wildtype diffuse astrocytic tumors that did not fulfill these GBM criteria-including WHO grade 2 and grade 3 diffuse astrocytomas-were collectively designated as "non-GBM *IDH*-wt gliomas" for analytical purposes in this study. These non-GBM cases were further stratified by WHO grade in subgroup analyses.

**Transcriptomic analysis**

Tumor specimens were selected from six patients with primary *IDH*-wt glioblastoma (three with *TP53*-wt and three with *TP53*-mut), matched for age, sex, and tumor location to minimize confounding. All six samples were processed in a single batch for RNA extraction, library construction, and sequencing. Raw reads were filtered and trimmed using fastp (v0.20.0), and aligned to the GRCh38 reference genome with STAR (v2.7.9a). Gene counts were quantified using featureCounts (v2.0.3). Differential expression analysis was performed using DESeq2 (v1.38.3), with genes considered significantly differentially expressed if |log2(fold change)| ≥ 1.0 and false discovery rate (FDR) adjusted P < 0.05, using the Benjamini-Hochberg method. KEGG pathway enrichment was performed using clusterProfiler (v4.6.0) with FDR < 0.05 as the significance threshold.

**Metabolomic analysis**

Non-targeted metabolomic profiling was performed on 12 frozen tumor tissues (six TP53-wt and six TP53-mut, matched for age and WHO grade) using ultra-high-performance liquid chromatography-tandem mass spectrometry (UHPLC-MS/MS) on a Thermo Q-Exactive Orbitrap system. Data acquisition and processing were conducted by BGI Genomics (Shenzhen, China). Metabolites were identified by matching MS/MS spectra against the Human Metabolome Database (HMDB) and METLIN. Differential metabolites were selected based on variable importance in projection (VIP) ≥ 1.0 from orthogonal partial least-squares discriminant analysis (OPLS-DA), combined with |log2(fold change)| ≥ 1.0 and P < 0.05 (Student's t-test). FDR correction was applied using the Benjamini-Hochberg method for multiple comparisons. Pathway enrichment was analyzed using MetaboAnalyst 5.0.

**Multivariable Cox regression analysis**

Multivariable Cox proportional hazards regression models were employed to evaluate the independent prognostic value of each variable for OS. Covariates included in the multivariable models were selected a priori using a literature-driven approach combined with clinical plausibility. Specifically, we referred to well-established prognostic factors in *IDH*-wildtype glioma and incorporated age, tumor category, extent of resection, chemotherapy, radiotherapy, and KPS score into the final model. All covariates were entered simultaneously using the enter method. Missing data were handled using complete-case analysis. All statistical analyses were performed using R software (version 4.3.1) and SPSS (version 27.0), and a two-sided *P*-value < 0.05 was considered statistically significant.

**PDO drug sensitivity assays**

For each PDO preparation, drug sensitivity was assessed using eight concentration gradients (ranging from 0.01 to 100 μM) for each tested agent. Three independent biological replicates were performed for each PDO. PDO viability was measured using the CellTiter-Glo 3D assay after 72 hours of drug exposure. IC50 values were calculated by nonlinear regression using a log(inhibitor) vs. normalized response model with variable slope (GraphPad Prism 8.0).

| **Table S1. Clinicopathological features of primary glioma patients in NFH cohort.** | |
| --- | --- |
| **Features** | **Cases (n=237)** |
| Age, years | 46.0±16.5 |
| Gender |  |
| Male | 144 (60.8%) |
| Female | 93 (39.2%) |
| Height (cm) | 164.8±7.6 |
| Body Mass Index (BMI, kg/m^2^) |  |
| BMI＜18.5 | 15 (6.3%) |
| 18.5≤BMI＜25 | 136 (57.4%) |
| BMI≥25 | 66 (27.8%) |
| NA | 20 (8.4%) |
| Karnofsky performance status (KPS) |  |
| KPS＜70 | 9 (3.8%) |
| KPS≥70 | 228 (96.2%) |
| Headache symptom |  |
| Yes | 116 (48.9%) |
| No | 121 (51.1%) |
| Epilepsy |  |
| Yes | 54 (22.8%) |
| No | 183 (77.2%) |
| Tumor location |  |
| Supratentorial | 207 (87.3%) |
| Infratentorial | 30 (12.7%) |
| Contrast-enhanced T1-weighted imaging |  |
| Yes | 193 (81.4%) |
| No | 44 (18.6%) |
| Increased intracranial pressure |  |
| Yes | 108 (45.6%) |
| No | 129 (54.4%) |
| Extent of resection |  |
| Biopsy | 2 (0.9%) |
| Partial resection | 29 (12.2%) |
| Subtotal and total resection | 206 (86.9%) |
| *MGMT* promoter methylation |  |
| Methylation | 83 (35.0%) |
| Non-methylation | 113 (47.7%) |
| NA | 41 (17.3%) |

NA, not available.

**Figure S1**. The histological spectrum of *IDH*-wt glioma (n=237).

**Figure S2.** Forest plot of Cox multivariate analysis for the clinical outcomes in non-GBM subgroup.

**Figure S3**. Kaplan-Meier survival curves for outcomes between *TP53*-wt and *TP53*-mut (VAF<10%) patients.

**Figure S4.** The alteration frequency with mutation type and mutation site of *TP53* in 237 *IDH*-wt gliomas.
